# Relevance of Individual Data When Assessing the Gastrointestinal Nematode Infection Level, Nutritional and Productive Variables in a Tropical Farm Context: The Median Isn’t the Message

**DOI:** 10.3390/ani14040603

**Published:** 2024-02-12

**Authors:** Gabriel Andrés Ortíz-Domínguez, Pedro Geraldo González-Pech, Juan Felipe de Jesús Torres-Acosta, Javier Ventura-Cordero, Juan Villalba, Carlos Alfredo Sandoval-Castro

**Affiliations:** 1 Facultad de Medicina Veterinaria y Zootecnia, Universidad Autónoma de Yucatán, Km 15.5 Carretera Mérida-Xmatkuil, Mérida C.P. 97315, Yucatán, Mexico; gabriel.ortiz.mvz@gmail.com (G.A.O.-D.); pedro.gonzalez@correo.uady.mx (P.G.G.-P.); tacosta@correo.uady.mx (J.F.d.J.T.-A.); 2 Facultad de Ciencias Agropecuarias, Universidad Autónoma de Campeche, Calle 53 S/N, Col. Unidad, Esfuerzo y Trabajo #2, Escárcega C.P. 24350, Campeche, Mexico; venti19@hotmail.com; 3 Department of Wildland Resources, Utah State University, Logan, UT 84322, USA; juan.villalba@usu.edu

**Keywords:** gastrointestinal nematodes, farming conditions, productive parameters, body condition, Criollo goats

## Abstract

**Simple Summary:**

Under field conditions, interactions occur between animals, parasites and the environment. Usually, only 30% of goats (over a dispersed distribution) display high gastrointestinal nematode (GIN) burdens. Hence, focusing on individual infection would allow better management of the impact of GINs. As acclaimed evolutionary biologist and natural historian S.J. Gould (1941–2002) once emphasized: “The Median Isn’t the Message”. The relationship between individual and herd GIN infection levels, nutritional and productive performance and anemia parameters in a tropical farm context were evaluated. Fifty-four female goats browsing the tropical forest (supplemented with balanced feed and chopped grass) were used. Measurements were obtained every 15 days (six samplings during the study); likewise, in each sampling time, the fecal samples were obtained in the morning [AM] and afternoon [PM] to assess the GIN burdens (eggs per gram of feces). The individual AM and PM values were similar and highly correlated at each sampling time. The nutritional and productive status fluctuated over time, although the median GIN burden of the flock was relatively constant. Nevertheless, the individual GIN burden differed when assessed at 30 d intervals; thus, these sampling points can be considered independent. Higher GIN burdens were associated with low body condition and low hematocrit. Moreover, the marked fluctuation of live weight should warn that nutrition is highly dynamic and could have a more important effect than GIN burden on the performance of goats under farm conditions in the tropics.

**Abstract:**

We evaluated the relationship between individual and herd GIN infection level, nutrition, production performance and anemia parameters in a tropical farm context. Fifty-four female goats were monitored to assess their body condition score (BCS, nutritional status indicator), live weight (LW) and LW gain (LWG, both used as production level indicators), FAMACHA© and hematocrit (HT, both used as anemia indicators). Goats browsed for 4 h in a tropical forest and received balanced feed and chopped grass. The eggs per gram of feces (EPG) indicated the GIN burden, with fecal samples obtained at 7:00 (AM) and 15:00 h (PM.) from each goat at six sampling points during the study. The variables and their relationship with GIN burdens were analyzed using Kruskall–Wallis, ANOVA and Friedman tests and Spearman correlations. The fecal samples obtained in the AM and PM can be equally representative of parasitic burdens (similar and highly correlated). However, the EPG of individual goats from periods of 30 days apart can be considered independent. The BCS and LWG varied between sampling times (*p* < 0.05), whereas EPG, LW and HT did not (*p* > 0.05). The GIN burden was negatively correlated with HT and BCS (−0.21, *p* = 0.01 for each one). The individual pattern of infection demonstrates the true impact of GINs on their hosts. Additionally, feeding and nutritional status may present important variations influencing the performance of the goats more than the impact of GINs under the farm conditions of the present study. However, GIN infection contributed to the variation in goat health and productivity in this tropical farm.

## 1. Introduction

Gastrointestinal nematode (GIN) infections are considered a threat to the production and health of grazing goats at a global scale [1,2]. Research to evaluate the impact of GIN infections or to evaluate different control methods is based on pen and field studies. Pen studies can control many variables that can affect GIN infections. Meanwhile, field studies under farm conditions are better to consider the complex relationship between the hosts (i.e., goats), the parasites (GIN species) and their environment. Field studies allow us to describe the build-up phenomenon, which represents the gradual and accumulative establishment of a given GIN population in the animals of a herd during a grazing season [3]. Consequently, in tropical and subtropical climates, the worm burden of goats browsing during the start of the rainy season would be low when the paddocks typically show a low GIN infectivity level. Meanwhile, at the end of the rainy season, the infectivity of the paddock and GIN burden of animals could be high [4]. The latter means that under natural grazing conditions, herds are constantly challenged at different intensities by infective GINs and this variation should be considered when investigating parasitism in small ruminants. Another feature is the over-dispersed distribution of GIN infections within the flock, which determines that in most flocks in open grazing conditions, approximately 30% of the goats show high GIN burdens [5,6]. Thus, during field trials, sampling a high number of individuals will be needed to find the few animals displaying medium to high GIN burdens. A recurrent question during the planning of parasitological studies using grazing/browsing animals is: how frequently the flock should be sampled. In the case of a full expression of the build-up phenomenon, gradual increases in GIN burden in the flock will lead to autocorrelated sampling points in time because all animals will increase their infection level. Nevertheless, changing conditions in the nutritional status could influence such a build-up. For example, sheep supplemented with an extra energy source are more likely to maintain GIN burden at moderate levels as the rainy season progresses [7]. The opposite effect could be present in animals with a sub-optimal nutritional level. Also, the level of production demanded from animals could disrupt the “normal” build-up of GIN infection in a herd, as reported for goats with high milk production, which have a higher risk of showing higher burdens of GIN parasites than lower-production goats [8]. Thus, it is possible that the nutritional status or the productive level of each goat affects its own gradual increase in GIN infection (build-up) and possibly causes a low correlation between sampling points over time.

Many authors use the count of eggs per gram of feces (EPG) as a standard method for evaluating the level of GIN infection [5,9,10]. Nevertheless, the strength of this test relies on variables such as the parasite species present, animal factors such as diarrhea or constipation and factors related to sample handling, for example, stool consistency, poor handling of specimens or the collection schedule [11]. In some parasitological studies, a common practice is sampling the same animal twice on the same day, a first sample of feces in the morning and a second during the afternoon, to calculate a mean value from both counts of EPG [9,12]. A recent pen study with young goats showed a strong correlation between the EPG values of samples taken in the morning and the afternoon [13]. Nevertheless, there is no information available on adult goats at browse for whom a well-established immunity could have an effect on their GIN excretion pattern. Thus, under on-farm conditions, the complexity and dynamic relationship between nutrition, productive performance and GIN infection deserves more research. In recent years, pooled samples have become more commonly used due to the popularity of automated FEC systems [14]. Furthermore, some authors suggest treating all the animals in the herd when a large proportion (>10%) of the herd are found to have FAMACHA© scores of 4 and 5 (anemic) [15]. This paper focuses on whether it is possible to gain adequate insight into the worm burden and the need for intervention for tropical goats by looking at mean or median values versus individual parameters for assessment. This study evaluated the relationship between the individual and flock GIN infection levels at different time points and the nutritional, productive performance and anemia parameters in a tropical farm context.

## 2. Materials and Methods

### 2.1. Farm Handling and Study Area

The study was performed at the small ruminant farm of the Faculty of Veterinary Medicine and Animal Science, Universidad Autónoma de Yucatán (FMVZ-UADY) (19°30′ N, 87°30′ W). The farm had a flock of 54 goats and 17 ewes. All the animals browsed for 4 h (from 7:00 to 11:00 AM.) in a tropical deciduous forest (TDF), obtaining almost 60% of their metabolizable energy (ME) requirements, with an excess of crude protein (108.2%) [16]. After daily grazing, goats were housed in their respective resting pens according to the usual farm management practices and received ~300 g (on a DM basis) of a balanced feed with 14% crude protein (CP) and 1.8 MCal ME/kg DM, except in the last week of November due to internal farm management. Goats also received ~50 g of fresh chopped grass (*Pennisetum purpureum*) with ~6% CP during the entire study period. The chopped grass was obtained from a non-grazed paddock. Animals did not have access to grazing materials when they were kept inside their pen. Water was available *ad libitum* in each resting pen.

The climate of the region is hot subhumid tropical with rainfall mainly during the summer (AW_0_). The average annual rainfall and relative humidity reach 940 mm and 72%, respectively. Nevertheless, the period between August and November is considered part of the rainy season. During that period of the year, the climatological conditions facilitate the survival of infective L_3_ larvae of GINs in the soil and the vegetation of the paddocks, but the number of L_3_ tends to be higher in November [4]. Adult goats browsing the TDF in the study region are expected to carry mixed infections mainly composed of *Haemonchus* spp. (44%), *Oesophagostomum* spp. (30%) and *Trichostrongylus* spp. (26%) [17]. Therefore, the GIN burden of experimental animals was acquired during daily grazing. The present study was performed from November 2021 to January 2022.

### 2.2. Experimental Animals and Variables Measured

**Figure 1:**
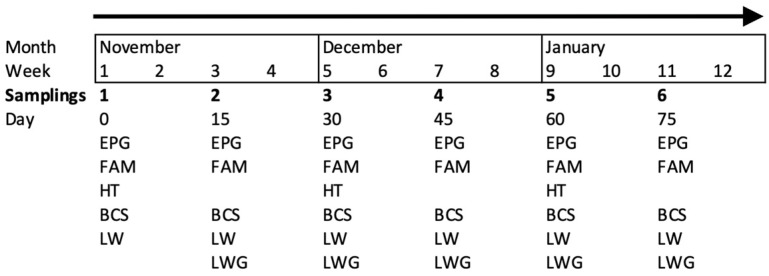
Sampling organization of the studied variables: egg count per gram of feces (EPG), coloration of the mucosa (FAM, FAMACHA©), body condition score (BCS), live weight (LW) and live weight gain (LWG, only five times) assessed every 15 days and hematocrit (HT) assessed monthly in adult goats browsing tropical deciduous forest.

Fifty-four adult goats were individually identified with numbered collars. Goats older than one year of age were included in the study and were at different physiological stages (either pregnant or non-pregnant). Most goats had >2 years of browsing experience at the TDF. The goats were distributed in six resting pens according to the handling scheme of the farm. All the animals were sampled six times (once every 15 days), except for the hematocrit (HT), which was assessed once each month (Figure 1).

The following variables were considered:

(i) Parasitological status. Fecal samples were obtained twice on each sampling day at 7:00 h (AM samples) and 15:00 h (PM samples) using plastic bags directly from the rectum of individual animals. The modified McMaster technique was used to count the eggs per gram of feces [18]. For this technique, two grams of feces were weighed and mixed with 28 mL of sugar flotation solution (1.28 density). Each egg counted represented 50 eggs per gram. On a monthly basis, a composite sample of individual goats’ feces from the different groups was used to prepare fecal cultures (maintained for five days at 27 °C). After six days of incubation, the L_3_ were recovered with the Baerman technique [19]. The L_3_ were identified using a microscope following specific morphological keys [20].
(ii) FAMACHA© score. This system was used to evaluate the color of the palpebral mucous membrane when compared to a color chart with 5 categories from pink to white [21]. When using FAMACHA©, scores of 4 and 5 to identify anemic goats. It has shown a specificity of 84% and a sensitivity of 60% [5].
(iii) Hematocrit (HT). A 3 mL blood sample was obtained directly from the jugular vein into EDTA tubes that were refrigerated until processing. The HT was determined using the micro-hematocrit method [22]. The anemia cut-off point was 21% [5].
(iv) Body condition score (BCS). The BCS was used as an indirect indicator of the nutritional status of goats. The methodology was performed as described by Honhold et al. [23], evaluating the amount of fat and muscle felt on key points of the lumbar column of goats. Scores ranged from 1 to 4.5 considering midpoints, where 1 was considered emaciated and 4.5 was considered very fat. A single trained technician measured each goat at the different sampling times.
(v) Assessment of the production level. The live weight (LW) and LW gain (LWG) between sampling times (six sampling events for LW and five for LWG, Figure 1) were employed. For these variables, all the animals were fasted for 12 h before weighing.

### 2.3. Statistical Analysis

The normal distribution of the variables was determined using the Shapiro–Wilk normality tests, as well as their respective tests of variance homogeneity (Levene tests).

To determine the differences between sampling points, the means for the anemia variables (FAMACHA© and HT) and medians for EPG were compared between the sampling points (days 0, 15, 30, 45 and 60) using Kruskal–Wallis tests for each of the variables. Additionally, a Student *t*-test served to compare all the EPG values obtained in the morning (AM) against those obtained in the afternoon (PM), irrespective of the sampling point. The parametric assumptions for the EPG data were confirmed prior to the analysis.

Respective Spearman correlation analyses were performed to identify the association between EPG values at different sampling points, including comparisons 15, 30, 45, 60 and 75 days apart.

To evaluate the independence of the EPG values of individual goats between sampling points, a Friedman test with its respective post hoc pairwise Wilcoxon tests was used to compare the sampling points. In addition, the position reached by each goat in the EPG data distribution (quartile 1, 2 or 3) at every sampling point was recorded and was compared to subsequent sampling points to identify (a) any increase or decrease in EPG by one quartile position (i.e., from Q1 to Q2, from Q2 to Q3 or the opposite) and (b) any increase or decrease in EPG by two quartile positions (i.e., from Q1 to Q3 or the opposite).

To determine the differences between sampling points for the nutritional variables (BCS) and productive variables (LW and LWG), means were compared between the sampling events (days 0, 15, 30, 45 and 60) using respective ANOVA tests for each of the variables. The effect of pregnancy (yes or no) and age (1, 3, 4, 5, 6, 7, 8 and 9 years) of the study goats on the LWG was not significant according to the ANOVA (*p* = 0.138 and *p* = 0.976, respectively).

Furthermore, respective Spearman correlation analyses were performed to identify the association between EPG values and the different nutritional, health and production variables. The different correlations included the data for each animal from days 0, 15, 30, 45, 60 and 75.

All the statistical analyses were performed with the Minitab^®^ 19.1, Minitab^®^ statistical software, Minitab^®^ Statistical Software Inc., Pennsylvania, PA, USA [24] with a significance level of *p* ≤ 0.05.

## 3. Results

### 3.1. Natural GIN Infection of Goats at Different Sampling Points

The medians (Q1 and Q3) of EPG excretion for S2, S3, S4, S5 and S6 were 500.0, (150.0 and 1025.0), 512.5 (250.0 and 856.2), 550.0 (193.7 and 837.5), 625.0 (375.0 and 1281.2) and 675.0 (318.7 and 1425.0), respectively. Non-significant differences were observed between sampling points (*p* = 0.065). The third-stage larvae identified from the group coprocultures performed in each calendar month were dominated by *Haemonchus* spp. (28.6–51.6%), followed by *Oesophagostomum* spp. (23.9–37.6%) and *Trichostrongylus* spp. (20.9–23.7%). The presence of small quantities of *Strongyloides* spp. eggs and larvae was evident at each sampling point.

Concerning the time of day when the fecal samples were obtained, a high correlation was found between the AM and PM mean EPG values of the different sampling times (r = 0.819, *p* < 0.001). When the AM and PM samples from all the events were considered, the correlation was also high (r = 0.804, *p* = 0.054). Furthermore, the Student *t*-test showed no differences (*p* = 0.362) between the mean EPG of all the samples obtained in the morning (AM) (977 ± 222 EPG) and the afternoon (PM) (860 ± 200 EPG).

### 3.2. Values of HT and FAMACHA© during the Study

The mean value of FAMACHA© 3 was maintained for the different sampling points. In the case of the HT values, the median was 25.4% on S1 and S2, 26.0% on S3 and S4, and 26.5% on S5. Consequently, a similarity was found between the sampling points for the mean values of FAMACHA©, as well as for those of HT (*p* > 0.05).

### 3.3. Independence of the EPG Values of Individual Goats between Sampling Points

The correlation between the EPG values of goats sampled 15, 30, 45, 60 and 75 days apart are shown in Table 1. The Spearman correlations (r^s^) showed highly significant associations between the data of consecutive sampling points (r^s^ = 49 to 66%; *p* < 0.001). Sampling points obtained 30 days apart showed weaker associations (r^s^= 34 to 54%; *p* < 0.01). For the sampling points obtained 45 and 60 days apart, their association varied from 33 to 50% (*p* < 0.05).

The results of Friedman tests indicated that the position of animals ranked according to their EPG values was significantly different (*p* value = 0.002) between sampling points. A post hoc pairwise Wilcoxon test showed differences for sampling points S1 and S2 versus S4 and S6 (Table 2). Meanwhile, S3 was different from S4, S5 and S6.

**Figure 2:**
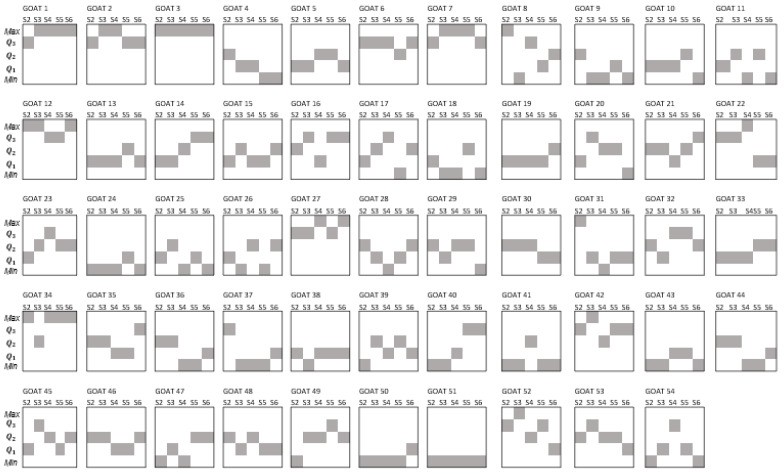
Individual quartile position (shaded boxes) according to the distribution of EPG values of GIN infection of goats during the same five sampling points.

Figure 2 displays the quartile position that individual goats showed according to the distribution of the EPG values during the experimental period (sampling points 1 to 5). In that figure, it is evident that few animals remained in the same quartile position. Up to 41% of the individuals changed their quartile position two times during sampling points one to five. Among the rest of the flock, 28% of the animals had three changes over time and 19% changed their quartile position four times. On the other hand, 9% changed once and 4% showed no change in their quartile position.

As shown in Figure 2, the quartile position of individuals was variable. Sometimes such variation/change consisted of an increase in their EPG value with respect to their former sampling (for example, passing from Q1 to Q3 of goat 45 at s1 and s2, respectively), while in other cases it showed a decrease (for example, moving from Q3 to Q1 of goat 52 from s4 to s5). Concerning the increments in the quartile position, forty-four goats showed increments of one quartile position, fifteen goats displayed increments of two quartile positions and one goat showed an increment of three quartile positions. Decrements of one quartile position were displayed by thirty-three goats, sixteen goats showed decrements of two quartile positions, five animals had decrements of three quartile positions and one goat showed a decrement of four quartile positions.

### 3.4. Pattern of Nutritional and Productive Performance Variables

**Figure 3:**
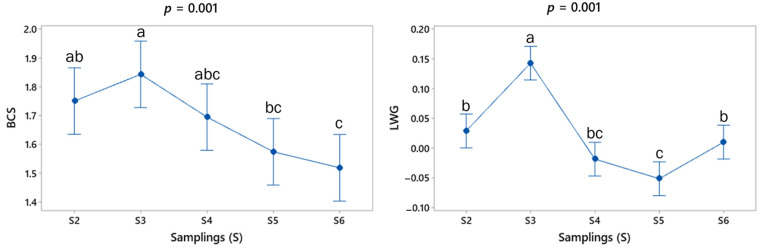
Mean (±SE) values (blue spots) at each sampling point for body condition score (BCS, points) and live weight gain (LWG, Kg/d) of adult goats grazing in the tropical deciduous forest. The respective *p* values for comparisons between sampling points are shown at the top of each graph. ^a,b,c^ Means with different letters between each period differ significantly (*p* < 0.05).

The BCS results show a mean value (min–max) of 1.5 (1–2.5) during the experimental period. Figure 3 shows that BCS presented a significant difference between sampling points S3, S5 and S6.

Concerning the indirect variables of production level, the LW mean values are given in kg (± standard error) for S1 (37.3 ± 7.88), S2 (37.7 ± 7.78), S3 (39.7 ± 8.13), S4 (39.5 ± 8.23) and S5 (38.8 ± 7.99). The pattern of mean values of LWG in the sampling points is shown in Figure 3. While the LW was similar during the study period (*p* = 0.363), some significant differences were found in the mean value of LWG (*p* < 0.05), showing LW gains (days 30 and 75) and losses (days 45 and 60) (Figure 3).

### 3.5. Link between GIN Infection Level, Nutritional, Productive and Anemia Variables

The significant correlations between GIN level measured as EPG excretion and the study variables evaluated 15 days apart are shown in Table 3. The EPG was negatively correlated with BCS and HT (*p* < 0.001). Furthermore, a positive association was found between EPG and FAMACHA© score (*p* < 0.05; Table 3).

## 4. Discussion

### 4.1. Natural GIN Infection of Goats at Different Sampling Points

In the present study, the GIN infection at the flock level measured as the number of eggs excreted per gram of feces (EPG) reached medians from 500 to 675 EPG during the experimental period, suggesting that the flock parasite burden remained stable and at similar levels over time. This pattern confirms the low and stable GIN infection shown by adult goats browsing low deciduous forests reported by Novelo-Chi et al. [25] and is highlighted as a characteristic of the Criollo goats of this region [5]. When maximum EPG values were investigated, the level found in the present study was from 1325 to 2750, which can be considered similar to the report of a contemporary study with adult goats browsing the same area (~2950 EPG) [17]. In contrast, this level was low compared to the maximum of 9800 EPG reported in a survey of 103 goat flocks from smallholders [5]. Thus, the infection level of the group of goats in the present study can be considered a mild infection. A similar pattern has been reported in goats under temperate conditions facing an artificial and natural challenge with *Teladorsagia circumcincta* and *T. colubriformis* [26]. In that study, the GIN infection level was well correlated between sampling moments for goats with high or low EPG excretion. Also, the study by Chartier and Hoste [27] in naturally infected goats showed that the EPG values between samplings were stable and had good repeatability.

The group EPG values in this study remained stable over time, contrasting with earlier reports obtained with tracer kids grazing the same type of vegetation (TDF) during the rainy season [4]. Those authors reported a build-up of infection during the rainy season. Such a build-up did not occur in the present study with a flock of adult goats, probably due to the following aspects: (a) while the tracer kids fail to show an immune response against GIN infections, adult goats have a competent immune system that can help them avoid further L_3_ establishment in the hosts [28]; (b) the existing adult worm population may block further establishment of incoming L_3_ through competition between larvae of the same species [29] or as a consequence of a hypersensitivity reaction known as the self-cure phenomenon, which is caused by previous larvae development that generates the expulsion of most of the worms in the abomasum when they are artificially or naturally infected [30]; (c) there can be an indirect effect of animal experience, leading goats to avoid plants near the feces [31] or a shift towards high-strata plants during the morning [17]; and (d) adult goats grazing TDF seek to balance the excess of degradable protein in the rumen by consuming plants with secondary compounds, but the latter can induce a prophylactic antihelminthic effect [10,12].

According to the present findings, the fecal cultures are consistent with the species of GIN identified for the study area in the rainy season in previous experiments using tracer kids raised free of infection from birth [4,32]. Those studies showed the presence of *Haemonchus contortus*, *Trichostrongylus colubriformis* and *Oesophagostomum columbianum*. Furthermore, the mixed infections and worm burdens found in the present survey were consistent with recent reports for adult goats browsing the tropical forest in México [10,17]. The presence of *Strongyloides* spp. in goats is also common for the ecosystem of the hot tropical conditions of Yucatán, México [33].

In the present study, we found that the EPG values from fecal samples obtained in the morning (AM) and the afternoon (PM) were not significantly different (*p* > 0.05). The effect of the time of the day on the GIN egg excretion of goats has not been commonly investigated in goats. To the best of our knowledge, there is only a single study that compared the EPG of kids artificially infected with *H. contortus* [13]. That study recorded the EPG of ten kids for 36 days and reported a similar EPG result in the morning compared to the afternoon. The results of the present study were obtained using more animals, all of them adults, under grazing/browsing conditions and on repeated weeks, which allowed a more robust result. In this manner, we confirmed that adult goats can be sampled in the morning or the afternoon of the same day, as there were no differences in the EPG between samples taken at different moments. This contributed to justifying that sampling once a day (whether AM. or PM) is equally valid as sampling twice a day. This represents an advantage for the welfare of experimental animals during parasitological studies.

### 4.2. Independence of the EPG Values of Individual Goats between Sampling Points

A significant correlation between the EPG values on different days was observed (Table 1). However, the coefficient of correlation was considerably higher for samples obtained 15 days apart (0.66 for S5 and S6, for example), while it only reached 0.33 to 0.38 in samples obtained 30 or 45 days apart (S3 and S4, S4 and S6, S1 and S4, S3 and S6; Table 1). The latter suggests that even when there is a significant correlation between the EPG values at different sampling points, the variation increased as the sampling points were more distant. The latter was confirmed in the Friedman test, where the ranks of goats according to their EPG values significantly differed in most of the sampling periods (Table 2). The Wilcoxon test showed that the ranks at S1 (day 0) and S2 (day 15) were similar, and both differed from S4 (day 45) and S6 (day 75). Meanwhile, the ranks at S3 (day 30) differed from that of S4, S5 and S6 (days 45, 60 and 75, respectively). Thus, when sampling more than 15 days apart, the EPG results tended to differ. These findings are similar to those reported by Vlassoff et al. [34], who mentioned that the EPG repeatability was higher between samples obtained at short time intervals (7–14 days apart), while longer periods tend to have poor repeatability.

In the present study, several goats changed their EPG rank position between sampling periods (Figure 2). As a result, different goats were in the high-EPG group at each sampling time and the same happened for the low-EPG group. These findings demonstrated that the individual EPG was dynamic, with changes in quartile position between sampling points in adult animals. The individual change in the EPG quartile rank was observed in this study because fecal samples were processed for all the goats in the study herd, and this is not conventionally performed for the control of GIN infections in a commercial flock. In the suggested targeted selective treatment schemes [5], fecal samples are obtained only from goats with poor BCS or pale mucosa. The latter results in a handful of individual samples every month, and there is no way to know what happens to the EPG of the non-sampled animals. However, there was a study performed with naturally infected hair sheep ewes grazing tropical pastures [35]. In that study, the ewes were sampled ten times over a period of 70 days. In that study, ewes were grouped according to the number of sampling points in which they had low or high EPG (6/10 occasions consecutive or non-consecutive) as resistant (29% of the ewes) or *susceptible* (17% of the ewes). In that study, 54% of the ewes changed quartile position on one or more occasions throughout the study, and these ewes were classified as *intermediate*. The latter suggests that, under natural infections with grazing animals, it is common to find changes in the quartile position of sheep. The latter is similar to the results of the present study with adult goats grazing the TDF.

A recent study by Mancilla-Montelongo et al. [36] reported that ~30% of adult goats naturally infected with GINs did not present infections above the established threshold (750 EPG) during 19 months of evaluation. For the remaining percentage (70%), feces were collected on more than one occasion (two to seven) throughout the study, resulting in EPG above the threshold and consequently, the use of a strategy for its control (supplementation or deworming). These results also suggest that the EPG excretion is not uniform over time. In the present study, the individuals who showed a reduction in their EPG could be considered resistant, while the goats that increased their EPG levels at one or more sampling points could represent susceptible animals. As mentioned above, these EPG variations could also be due to factors of the animal (premunity) or the environment (nutrients in the TDF, plant secondary compounds) [10,16]. The interaction among these factors will be described later.

The negative association between HT and EPG has been reported for tropical goats in México (r = −0.32; *p* < 0.001) [5]. Those same authors reported a poor correlation between the EPG values and the FAMACHA^®^ scores (r = 0.06; *p* > 0.05). Another study reported that ~1500 EPG was associated with FAMACHA© score 3 and >1020 EPG was associated with PCV < 19% for goats in temperate regions [37], while in tropical adult goats browsing the tropical forest, an HT between 27.2 and 28.8% was found when EPG levels were ~1050 EPG [17]. In the present study, the mean FAMACHA© score and mean HT remained stable and at normal values (*p* > 0.05) during the different sampling points despite the variable EPG values described above. However, the EPG data did show a significant negative correlation with HT, as well as a positive correlation with FAMACHA© scores (Table 3). Furthermore, eight goats showed a reduction in HT to below 22%, which is the threshold used to declare anemia [5,38], and most of these goats had >1000 EPG. Hence, the mean values at each sampling point could be deceiving when attempting to evaluate the impact of EPG values on the anemia threshold.

### 4.3. Nutritional Status and Its Association with GIN Infection Level

In the present study, the indicator of the nutritional status (BCS) showed low results (≤2) across all the sampling points. Meanwhile, the pattern of mean EPG values found suggested that the GIN infection was low to mild. Despite the latter, a significant negative correlation between EPG and the BCS was found (r = −0.216, *p* < 0.05; Table 3). The latter suggests a mild negative association between GIN infection and nutritional status. A deleterious effect of GIN infection on the BCS is commonly observed in temperate regions in goat breeds like Saaneen, Toggenburg and Chamoise [37]. Also, a negative relationship between GIN infection (at levels of 1603 EPG) and BCS has been reported for adult goats in a tropical region [39].

The low coefficients of correlation obtained (−0.216) in the present study suggest that the deficit in nutritional status was poorly explained by the EPG level. It has been proposed that evident changes in BCS, FAMACHA scores or HT values can only be observed with high GIN infection in goats browsing subtropical regions [40]. According to Coop and Holmes [41], the infection must reach levels >2400 EPG to cause haemonchosis. However, when the levels are not high, they do not cause significant losses in the economy of the animals in nutritional terms of body weight and BCS [42,43,44]. Thus, a low coefficient of correlation may suggest that factors different to GIN infection were present and could help to explain the negative association with BCS.

### 4.4. Productive Variables Not Associated with GIN Infection Level

In the present study, neither the LW nor the LWG was associated with the EPG excretion of goats (ns, Table 3). The LW of the experimental goats remained stable between sampling periods; this could be explained due to their age, as they were already in their adulthood [45]. Also, this result confirms the observations of Torres-Fajardo et al. [10], where the weight of goats with GIN infection at browsing remained stable.

The constant LW between sampling points that was observed contrasted with the LWG, which showed significant changes during the study. It is probable that some animals were gaining weight at some sampling points (LWG from days 30 and 75, Figure 3) and losing weight at other sampling points (LWG from days 45 and 60, Figure 3). Thus, the general performance observed as the median weight showed a stable pattern. Gaining and losing weight has been reported for adult goats naturally infected with GINs during the rainy season [25]. Nevertheless, those changes in the live weight are probably driven by the nutritional nature of the low deciduous forest. This ecosystem, with forage abundance during the rainy season, can result in an unbalanced (high protein but low energy content) diet for goats. The latter could lead to an extra energy cost of metabolizing the excess nitrogen, thus losing weight in the animals when the supplement is not appropriate in quantity or quality [16]. Furthermore, the farm management caused irregular feed intake in terms of quality and quantity due to changes in the number of grazing hours per day. Also, the quantity of supplementary feeding provided to each animal varied due to availability at the farm, with zero supplement during some weeks. In summary, the feeding management of the goats seemed to be more relevant than the GIN infection per se as a negative modifier of the nutritional and productive status of goats. These findings confirm previous results from a cafeteria trial suggesting that adult goats are more affected by nutrition than by GIN infection [9]. Even more, a recent study showed that the strategic use of supplementary feeding may be beneficial to enhance the nutritional status, and even reduce the EPG of adult goats in tropical field conditions [36]. Similarly, these results provided further support for the implementation of targeted GIN control strategies with a focus on individuals and not the flock.

## 5. Conclusions

A low to mild GIN infection was observed in adult goats browsing tropical deciduous forests during the rainy season. While the median EPG remained stable between sampling points when analyzed as a flock, individual goats showed variation in their quartile position when ranked according to their EPG excretion. The EPG level was negatively correlated with the BCS and HT and positively correlated with FAMACHA© scores. Nevertheless, the intensity of association between those variables was weak, suggesting that factors other than GIN infection could be involved. It is likely that under the conditions of a tropical farm with browsing adult goats, the variation in the nutritional status could cause a higher penalty to the animal economy than their GIN infection. Although at the flock level, the infection remains stable, the pattern of infection of each goat is dynamic. Furthermore, nutritional imbalance may be more relevant for BCS and LWG than the impact caused by GIN infection. However, the GIN infection contributed to the variation in goats’ health and productivity in this tropical farm.

## Figures and Tables

**Table 1:** Spearman correlation (r^s^) of individual gastrointestinal nematode fecal egg counts of naturally infected goats between sampling (S) times taken 15, 30, 45, 60 and 75 days apart.

Differences between Observation Days									
15		30		45		60		75	
Contrast	r^s^	Contrast	r^s^	Contrast	r^s^	Contrast	r^s^	Contrast	r^s^
S1–S2	0.582 ***	S1–S3	0.384 **	S1–S4	0.359 **	S1–S5	0.431 ***	S1–S6	0.451 **
S2–S3	0.547 ***	S3–S5	0.449 ***	S2–S5	0.500 ***	S2–S6	0.419 **		
S3–S4	0.499 ***	S2–S4	0.537 ***	S3–S6	0.337 *				
S4–S5	0.579 ***	S4–S6	0.336 **						
S5–S6	0.668 ***								

Significance level: * = *p* < 0.05, ** = *p* < 0.01, *** = *p* < 0.001.

**Table 2:** Comparison of nematode eggs per gram of feces (EPG) between different sampling points according to the respective post hoc pairwise Wilcoxon tests. The *p* values of paired comparisons (different sampling points) are shown.

	S1	S2	S3	S4	S5
S2	0.1877	-	-	-	-
S3	0.7498	0.3042	-	-	-
S4	0.0144 *	0.0195 *	0.0005 *	-	
S5	0.0679	0.0751	0.0036 *	0.6793	-
S6	0.0016 *	0.0085 *	0.0028 *	0.5094	0.2899

Significance level: * = *p* < 0.05

**Table 3:** Spearman correlations between the gastrointestinal nematode egg excretion level (expressed as eggs per gram (EPG)) and the productive, nutritional and anemia variables in adult goats in tropical conditions for three months.

Parameters	Variable	EPG	LW	LWG	BCS	FAMACHA©
Productive	Live Weight (LW)	−0.110 ns				
	LW Gain (LWG)	0.025 ns	−0.081 ns			
Nutritional	Body Condition Score (BCS)	−0.216 ***	0.423 ***	−0.009 ns		
Anemia	FAMACHA©	0.118 *	−0.118 *	0.004 ns	−0.472 ***	
	Hematocrit	−0.211 ***	0.237 ***	−0.006 ns	0.493 ***	−0.373 ***

Significance level: * = *p* < 0.05, *** = *p* < 0.001, ns = not significant.

## Data Availability

The authors can share the available data upon reasonable request.

## References

[1] Ares Annual State-of-the Art Report on Animal Health Research on IRC Priorities. European H2020. 5094086—18/10/2017. https://ec.europa.eu/research/participants/documents/downloadPublic?documentIds=080166e5b5d328a0&appId=PPGMS.

[2] Charlier J., Rinaldi L., Musella V., Ploegerd H.W., Chartier C., Rose Vineer H., Hinney B., von Samson-Himmelstjerna G., Băcescu B., Mickiewicz M. (2020). Initial assessment of the economic burden of major parasitic helminth infections to the ruminant livestock industry in Europe. Prev. Vet. Med..

[3] Sissay M.M., Uggla A., Walker P.J. (2007). Prevalence and seasonal incidence of nematode parasites and fluke infections of sheep and goats in eastern Ethiopia. Trop. Anim. Health Prod..

[4] Jaimez-Rodríguez P.R., González-Pech P.G., Ventura-Cordero J., Brito D.R.B., Costa-Júnior L.M., Sandoval-Castro C.A., Torres-Acosta J.F.J. (2019). The worm burden of tracer kids and lambs browsing heterogeneous vegetation is influenced by strata harvested and not total dry matter intake or plant life form. Trop. Anim. Health Prod..

[5] Torres-Acosta J.F.J., Pérez-Cruz M., Canul-Ku H.L., Soto-Barrientos N., Cámara-sarmiento R., Aguilar-Caballero A.J., Lozano-Argáes I., Le-Bigot C., Hoste H. (2014). Building a combined targeted selective treatment scheme against gastrointestinal nematodes in tropical goats. Small Rumin. Res..

[6] Sebatjane P.N., Njuho P.M., Tsotetsi-Khambule A.M. (2019). Statistical models for helminth faecal egg counts in sheep and goats. Small Rumin. Res..

[7] Retama-Flores C., Torres-Acosta J.F.J., Sandoval-Castro C.A., Aguilar-Caballero A.J., Cámara-Sarmiento R., Canul-Ku H.L. (2012). Maize supplementation of Pelibuey sheep in a silvopastoral system: Fodder selection, nutrient intake and resilience against gastrointestinal nematodes. Animal.

[8] Chartier C., Etter E., Hoste H., Pors I., Mallereau M.-P., Broqua C., Mallet S., Koch C., Massé A. (2000). Effects of the initial level of milk production and of the dietary protein intake on the course of natural nematode infection in dairy goats. Vet. Parasitol..

[9] Ventura-Cordero J., González-Pech P.G., Jaimez-Rodríguez P.G., Ortiz-Ocampo G.I., Sandoval-Castro C.A., Torres-Acosta J.F.J. (2017). Gastrointestinal nematode infection does not affect selection of tropical foliage by goats in a cafeteria trial. Trop. Anim. Health Prod..

[10] Torres-Fajardo R.A., Navarro-Alberto J.A., Ventura-Cordero J., González-Pech P.G., Sandoval-Castro C.A., Chan-Pérez J.I., Torres-Acosta J.F.J. (2019). Intake and selection of goats grazing heterogeneous vegetation: Effect of gastrointestinal nematodes and condensed tannins. Range. Ecol. Manag..

[11] Rodríguez-Vivas R.I., Cob-Galera L.A. (2005). Técnicas Diagnósticas en Parasitología Veterinaria.

[12] Ventura-Cordero J., González-Pech P.G., Jaimez-Rodriguez P.R., Ortiz-ocampo G.I., Sandoval-Castro C.A., Torres-Acosta J.F.J. (2018). Feed resource selection of Criollo goats artificially infected with *Haemonchus contortus*: Nutritional wisdom and prophylactic self-medication. Animal.

[13] Ngongeh L.A. (2017). Variation in faecal worm egg counts of experimentally infected goats and mice with time of day and its implications in diagnosis of helminthosis. J. Parasit. Dis..

[14] Cain J.L., Gianechini L.S., Vetter A.L., Davis S.M., Britton L.N., Myka J.L., Slusarewicz P. (2024). Rapid, automated quantification of *Haemonchus contortus* ova in sheep faecal samples. Int. J. Parasitol..

[15] Bath G., Malan F., van Wyk J. (2021). FAMACHA© information pamphlet and guide. WURMFUNDI What Works with Worms.

[16] Ventura-Cordero J., González-Pech P.G., Torres-Acosta J.F.J., Sandoval-Castro C.A., Tun-Garrido J. (2019). Sheep and goat browsing a tropical deciduous forest during the rainy season: Why does similar plant species consumption result in different nutrient intake?. Anim. Prod. Sci..

[17] Torres-Fajardo R.A., González-Pech P.G., Sandoval-Castro C.A., Ventura-Cordero J., Torres-Acosta J.F.J. (2019). Criollo goats limit their grass intake in the early morning suggesting a prophylactic self-medication behaviour in a heterogeneous vegetation. Trop. Anim. Health Prod..

[18] Bauer B.U., Pomroy W.E., Gueydon J., Gannac S., Scott I., Pfister K. (2010). Comparison of the FLOTAC technique with the McMaster method and the Baermann technique to determine counts of *Dictyocaulus eckerti* L1 and strongylid eggs in faeces of red deer (*Cervus elaphus*). Parasitol. Res..

[19] MAFF, Ministry of Agriculture, Fisheries and Food (1986). Helminthology. Manual of Veterinary Parasitological Laboratory Techniques.

[20] Van Wyk J.A., Mayhew E. (2013). Morphological identification of parasitic nematode infective larvae of small ruminants and cattle: A practical lab guide. Onderstepoort J. Vet. Res..

[21] Kaplan R.M., Burke J.M., Terrill T.H., Miller J.E., Getz W.R., Mobini S., Valencia E., Williams M.J., Williamson L.H., Larsen M. (2004). Validation of the FAMACHA eye color chart for detecting clinical anemia in sheep and goats on farms in the southern United States. Vet. Parasitol..

[22] Benjamin M.M. (1991). Manual de Patología Clínica en Veterinaria.

[23] Honhold N., Petit H., Halliwell R.W. (1989). Condition scoring scheme for Small East African goats in Zimbabwe. Trop. Anim. Health Prod..

[24] Minitab (2019). Minitab Statistical Software. Release 19.1.

[25] Novelo-Chi L.K., González-Pech P.G., Ventura-Cordero J., Torres-Acosta J.F.J., Sandoval-Castro C.A., Cámara-Sarmiento R. (2019). Gastrointestinal nematode infection and feeding behaviour of goats in a heterogeneous vegetation: No evidence of therapeutic self-medication. Behav. Process..

[26] Patterson D.M., Jackson F., Huntley J.F., Stevenson L.M., Jones D.G., Jackson E., Russel J.F. (1996). Studies on caprine responsiveness to Nematodiasis: Segregation of male goats into responders and non-responders. Int. J. Parasitol..

[27] Chartier C., Hoste H. (1997). Repeated infections with *Haemonchus contortus* and *Trichostronlylus colubriformis* in dairy goats: Comparison of resistant and susceptible animals. Parasitol. Res..

[28] Dilgasa L., Asrade B., Kasaye S. (2015). Prevalence of gastrointestinal nematodes of small ruminants in and around Arsi Negele Town, Ethiopia. Am.-Eurasian J. Sci. Res..

[29] LeJambre L.F., Ractliffe L.H. (1971). Seasonal change in a balanced polymorphism in *Haemonchus contortus* populations. Parasitology.

[30] Allonby E.W., Urquhart G.M. (1973). Self-cure of *Haemonchus contortus* infections under field conditions. Parasitology.

[31] Hutchings M.R., Kyriazakis I., Anderson D.H., Gordon I.J., Coop R.L. (1998). Behavioural strategies used by parasitised and non-parasitised sheep to avoid ingestion of gastrointestinal nematodes. Anim. Sci..

[32] Torres-Acosta J.F.J., Jacobs D.E., Aguilar-Caballero A., Sandoval-Castro C., May-Martínez M., Cob-Galera L.A. (2004). The effect of supplementary feeding on the resilience and resistance of browsing Criollo kids against natural gastrointestinal nematode infections during the rainy season in tropical Mexico. Vet. Parasitol..

[33] Mendoza-de-Gives P., Torres-Acosta J.F.J., Figueroa-Castillo J.A., Soberanes-Céspedes N., Mancilla-Montelongo M.G., Jasso-Villazul C.E., Neri-Orantes S., Rodríguez-Vivas R.I., Vega y Murguía C.A. (2023). Diagnostico y Control Sustentable de Nematodos Gastrointestinales en Ovinos y Caprinos en la Era de la Resistencia Antihelmíntica.

[34] Vlassoff A., Bisset S.A., McMurtry L.W. (1999). Faecal egg counts in Angora goats following natural or experimental challenge with nematode parasites: Within-flock variability and repeatabilities. Vet. Parasitol..

[35] Palomo-Couoh J.G., Aguilar-Caballero A.J., Torres-Acosta J.F.J., Magaña-Monforte J.G. (2016). Evaluation of different models to segregate Pelibuey and Katahdin ewes into resistant or susceptible to gastrointestinal nematodes. Trop. Anim. Health Prod..

[36] Mancilla-Montelongo G., González-Pech P.G., Miranda-Miranda D.Y., Castañeda-Ramírez G.S., Encalada-Aguilar P.A., Can-Celis A., Galera-Chan I.E., Ortíz-Domínguez G.A., Torres-Acosta J.F.J. (2023). Targeted treatment strategies for the control of gastrointestinal nematodes in a goat flock with anthelmintic resistant worm populations and poor nutrition. Small Rumin. Res..

[37] Scheuerle M., Mahling M., Muntwyler J., Pfister K. (2010). The accuracy of the FAMACHA-method in detecting aneamia and haemonchosis in goat flocks in Switzerland under field conditions. Vet. Parasitol..

[38] Johns J., Heller M. (2021). Hematologic Conditions of Small Ruminants. Vet. Clin. Food Anim..

[39] Wuthijaree K., Tatsapong P., Lambertz C. (2022). The prevalence of intestinal parasite infections in goats from smallholder farms in Northern Thailand. Helminthologia.

[40] Bessell P.R., Sargison N.R., Mirende K., Dash R., Prasad S., Al-Riyami L., Gammon N., Stuke K., Woolley R., Barbaruah M. (2018). The impact of anthelmintic drugs on weight gain of smallholder goats in subtropical regions. Prev. Vet. Med..

[41] Coop R.L., Holmes P.H. (1996). Nutrition and Parasite Interaction. Int. J. Parasitol..

[42] Haile A., Hassen H., Gatew H., Getachew T., Lobo R.N.B., Rischkowsky B. (2018). Investigations into nematode parasites of goats in pastoral and crop livestock systems of Ethiopia. Trop. Anim. Health Prod..

[43] Ramos-Bruno E., Sandoval-Castro C.A., Torres-Acosta J.F.J., Sarmiento-Franco L.A., Torres-Fajardo R., Chan-Pérez J.I., Ortiz-Ocampo G.I. (2021). Nitrogen retention in hair sheep lambs with a gradient of *Haemonchus contortus* infection. Vet. Parasitol..

[44] Ramos-Bruno E., Torres-Acosta J.F.J., Sarmiento-Franco L.A., Sandoval-Castro C.A. (2021). Metabolizable energy balance in hair sheep lambs artificially infected with *Haemonchus contortus*. Vet. Parasitol..

[45] Gougoulis D.A., Kyriazakis I., Fthenakis G.C. (2010). Diagnostic significance of behaviour changes of sheep: A selected review. Small Rumin. Res..

